# Baseline factors associated with early and late death in intracerebral haemorrhage survivors

**DOI:** 10.1111/ene.14238

**Published:** 2020-04-28

**Authors:** G. Banerjee, G. Ambler, D. Wilson, I. C. Hostettler, C. Shakeshaft, S. Lunawat, H. Cohen, T. Yousry, R. Al‐Shahi Salman, G. Y. H. Lip, H. Houlden, K. W. Muir, M. M. Brown, H. R. Jäger, D. J. Werring, the CROMIS‐2 collaborators, Louise Shaw, Kirsty Harkness, Jane Sword, Azlisham Mohd Nor, Pankaj Sharma, Deborah Kelly, Frances Harrington, Marc Randall, Matthew Smith, Karim Mahawish, Abduelbaset Elmarim, Bernard Esisi, Claire Cullen, Arumug Nallasivam, Christopher Price, Adrian Barry, Christine Roffe, John Coyle, Ahamad Hassan, Caroline Lovelock, Jonathan Birns, David Cohen, L. Sekaran, Adrian Parry‐Jones, Anthea Parry, David Hargroves, Harald Proschel, Prabel Datta, Khaled Darawil, Aravindakshan Manoj, Mathew Burn, Chris Patterson, Elio Giallombardo, Nigel Smyth, Syed Mansoor, Ijaz Anwar, Rachel Marsh, Sissi Ispoglou, Dinesh Chadha, Mathuri Prabhakaran, Sanjeevikumar Meenakishundaram, Janice O'Connell, Jon Scott, Vinodh Krishnamurthy, Prasanna Aghoram, Michael McCormick, Paul O’Mahony, Martin Cooper, Lillian Choy, Peter Wilkinson, Simon Leach, Sarah Caine, Ilse Burger, Gunaratam Gunathilagan, Paul Guyler, Hedley Emsley, Michelle Davis, Dulka Manawadu, Kath Pasco, Maam Mamun, Robert Luder, Mahmud Sajid, Ijaz Anwar, James Okwera, Julie Staals, Elizabeth Warburton, Kari Saastamoinen, Timothy England, Janet Putterill, Enrico Flossman, Michael Power, Krishna Dani, David Mangion, Appu Suman, John Corrigan, Enas Lawrence, Djamil Vahidassr

**Affiliations:** ^1^ Department of Brain Repair and Rehabilitation Stroke Research Centre UCL Queen Square Institute of Neurology and the National Hospital for Neurology and Neurosurgery London UK; ^2^ Department of Statistical Science University College London London UK; ^3^ New Zealand Brain Research Institute Christchurch New Zealand; ^4^ Haemostasis Research Unit Department of Haematology University College London London UK; ^5^ Lysholm Department of Neuroradiology and the Neuroradiological Academic Unit, Department of Brain Repair and Rehabilitation UCL Queen Square Institute of Neurology London UK; ^6^ Centre for Clinical Brain Sciences School of Clinical Sciences University of Edinburgh Edinburgh UK; ^7^ Liverpool Centre for Cardiovascular Science University of Liverpool and Liverpool Heart & Chest Hospital Liverpool UK; ^8^ Aalborg Thrombosis Research Unit Department of Clinical Medicine Aalborg University Aalborg Denmark; ^9^ Department of Molecular Neuroscience UCL Queen Square Institute of Neurology and the National Hospital for Neurology and Neurosurgery London UK; ^10^ Institute of Neuroscience & Psychology University of Glasgow Elizabeth University Hospital Queen Glasgow UK

**Keywords:** intracerebral haemorrhage, mortality, prognosis

## Abstract

**Background and purpose:**

The aim of this study was to determine whether early and late death are associated with different baseline factors in intracerebral haemorrhage (ICH) survivors.

**Methods:**

This was a secondary analysis of the multicentre prospective observational CROMIS‐2 ICH study. Death was defined as ‘early’ if occurring within 6 months of study entry and ‘late’ if occurring after this time point.

**Results:**

In our cohort (*n* = 1094), there were 306 deaths (per 100 patient‐years: absolute event rate, 11.7; 95% confidence intervals, 10.5–13.1); 156 were ‘early’ and 150 ‘late’. In multivariable analyses, early death was independently associated with age [per year increase; hazard ratio (HR), 1.05, *P* = 0.003], history of hypertension (HR, 1.89, *P* = 0.038), pre‐event modified Rankin scale score (per point increase; HR, 1.41, *P* < 0.0001), admission National Institutes of Health Stroke Scale score (per point increase; HR, 1.11, *P* < 0.0001) and haemorrhage volume >60 mL (HR, 4.08, *P* < 0.0001). Late death showed independent associations with age (per year increase; HR, 1.04, *P* = 0.003), pre‐event modified Rankin scale score (per point increase; HR, 1.42, *P* = 0.001), prior anticoagulant use (HR, 2.13, *P* = 0.028) and the presence of intraventricular extension (HR, 1.73, *P* = 0.033) in multivariable analyses. In further analyses where time was treated as continuous (rather than dichotomized), the HR of previous cerebral ischaemic events increased with time, whereas HRs for Glasgow Coma Scale score, National Institutes of Health Stroke Scale score and ICH volume decreased over time.

**Conclusions:**

We provide new evidence that not all baseline factors associated with early mortality after ICH are associated with mortality after 6 months and that the effects of baseline variables change over time. Our findings could help design better prognostic scores for later death after ICH.

## Introduction

Most research on outcomes following intracerebral haemorrhage (ICH) has focussed on short‐term mortality (within 6 months), reflecting the high rates of early death associated with this stroke subtype [1, 2]. Many factors associated with early mortality relate to ICH severity and this is reflected in prognostic scores that aim to predict outcome in the short term [3, 4, 5, 6, 7, 8, 9, 10, 11–3, 4, 5, 6, 7, 8, 9, 10, 11]. Policies of active acute management, including blood pressure lowering, prompt reversal of anticoagulation and neurosurgical referral, aim to improve prognosis in patients with ICH [12]. A better understanding of the factors that influence ‘late’ death following ICH might identify potentially modifiable risk factors that could improve long‐term outcomes [1].

Our aim was to evaluate whether early and late death are associated with different baseline factors in ICH survivors using data from the prospective multicentre CROMIS‐2 ICH study. We hypothesized that factors relating to the severity of the acute ICH would not be associated with death at later (beyond 6 months) time points.

## Methods

### Data availability statement

Analyses for the CROMIS‐2 study are ongoing; once all of these analyses are completed, the CROMIS‐2 Steering Committee will consider applications from other researchers for access to anonymized source data.

### Participants

We included adults from the CROMIS‐2 (Clinical Relevance of Microbleeds in Stroke) ICH study; full details of the study protocol have been published previously [13]. Further details are provided in the Supporting Information. The study was approved by the National Research Ethics Service (IRAS reference 10/H0716/61). Informed written consent was obtained for all participants.

### Outcomes

The outcome of interest for this project was time to death. Mortality notifications were received from NHS Digital (previously the Health and Social Care Information Centre) as detailed in the previously published study protocol [13]. NHS Digital is a national centralized body that collects data on health and social care in the UK; mortality data are derived from ‘hospital episode statistics’ (records of all NHS patient admissions) and information on registered deaths from the Office of National Statistics (death registration is a legal requirement in the UK).

Patients were censored at either 3 years following the ICH that resulted in study entry or at last available follow‐up for vital status (the time of the study’s last notification of deaths from NHS Digital, i.e. 31 October 2017), depending on which was earlier.

### Imaging

Brain computed tomography imaging was acquired acutely at the time of the index event as part of the patient’s routine clinical care. Further details are provided in the Supporting Information.

### Statistics

Statistical analysis was performed using Stata (version 15.1, StataCorp LLC, College Station, TX, USA). We dichotomized time following ICH into ‘early’ (before 6 months) and ‘late’ (after 6 months) periods. We used univariable Cox regression to calculate hazard ratios (HRs) for all baseline variables collected to review for associations during these two time periods. Variables where the 95% confidence intervals (CI) did not cross 1 were considered as statistically significant. To explore this further, the effect of each baseline variable was then allowed to vary linearly with time; further details are provided in the Supporting Information.

## Results

**Figure 1:**
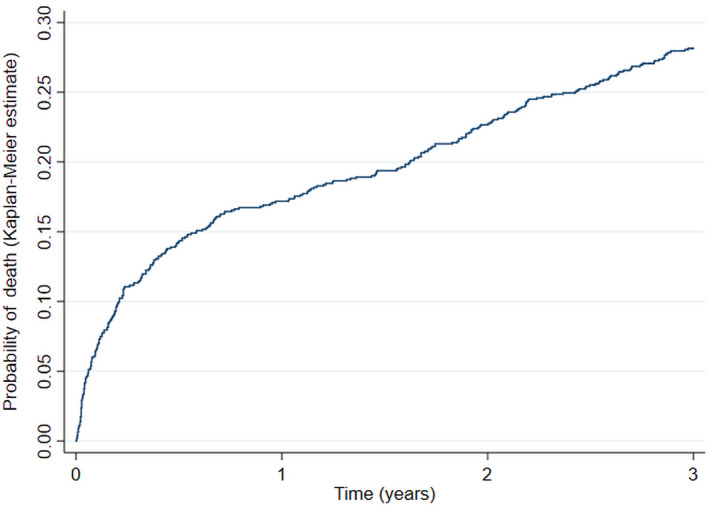
Unadjusted cumulative mortality curve.

All 1094 patients recruited to CROMIS‐2 ICH were included (Table 1). Follow‐up was for a total of 2613.48 patient‐years (median 3.00 years; interquartile range, 2.31–3.00 years). There were 306 deaths (absolute event rate, 11.7 per 100 patient‐years; 95% CI, 10.5–13.1 per 100 patient‐years; Fig. 1). The median time between the index ICH event and study entry was 4 days (interquartile range, 2–8 days).

**Table 1:** Baseline characteristics

	All	Alive	Early death (<6 months)	Late death (≥6 months)
*n*	1094	788 (72.0%)	156 (14.3%)	150 (13.7%)
Age (years)	73.3 ± 12.5	70.3 ± 12.4	81.1 ± 9.4	80.7 ± 8.5
Sex, male	628 (57.4%)	468 (59.4%)	78 (50.0%)	82 (54.7%)
Hypertension	718 (66.7%)	505 (65.3%)	114 (73.6%)	99 (66.9%)
Hypercholesterolaemia	467 (44.0%)	322 (42.0%)	71 (47.7%)	74 (50.3%)
Diabetes mellitus	202 (18.6%)	132 (16.9%)	38 (24.4%)	32 (21.6%)
Atrial fibrillation	375 (37.4%)	215 (30.1%)	81 (56.3%)	79 (55.2%)
Smoking (at time of ICH)	114 (10.8%)	94 (12.4%)	12 (8.0%)	8 (5.6%)
Pre‐existing cognitive impairment	251 (39.8%)	150 (34.3%)	54 (50.5%)	47 (54.7%)
Previous cerebral ischaemic event	241 (22.9%)	149 (19.5%)	44 (30.1%)	48 (33.8%)
Previous ICH	46 (4.3%)	28 (3.6%)	10 (6.7%)	8 (5.6%)
Pre‐event mRS score	0 (0–1)	0 (0–1)	1 (0–3)	1 (0–2)
*APOE* ε2, presence	189 (20.7%)	138 (20.8%)	31 (26.5%)	20 (15.3%)
*APOE* ε4, presence	256 (28.1%)	196 (29.5%)	24 (20.5%)	36 (27.5%)
Medications				
Antiplatelet use prior to ICH	267 (24.6%)	193 (24.7%)	38 (24.5%)	36 (24.2%)
Anticoagulant use prior to ICH	436 (40.1%)	261 (33.4%)	86 (55.5%)	89 (59.3%)
Antiplatelet use at discharge	65 (6.4%)	46 (6.2%)	8 (6.2%)	11 (7.8%)
Anticoagulant use at discharge	113 (10.7%)	78 (10.2%)	14 (9.5%)	21 (14.5%)
Clinical features at study entry				
GCS score	15 (14–15)	15 (14–15)	14 (11–15)	15 (14–15)
NIHSS score	7 (3–13)	6 (3–11)	14 (7–19)	6 (3–12)
Imaging features at study entry				
Lacunes, presence	98 (9.0%)	69 (8.8%)	15 (9.6%)	14 (9.3%)
Van Swieten score (WMC)	0 (0–2)	0 (0–2)	1 (0–3)	2 (0–3)
ICH location				
Infratentorial	99 (9.1)	69 (8.8)	12 (7.7)	18 (12.0)
Deep	546 (50.0)	398 (50.6)	69 (44.2)	79 (52.7)
Lobar	447 (40.9)	319 (40.6)	75 (48.1)	53 (35.3)
ICH volume				
<30 mL	886 (85.9)	655 (89.0)	106 (70.7)	125 (85.6)
30–60 mL	99 (9.6)	60 (8.2)	24 (16.0)	15 (10.3)
>60 mL	47 (4.6)	21 (2.9)	20 (13.3)	6 (4.1)
Intraventricular extension	301 (27.7)	183 (23.4)	68 (43.6)	50 (33.6)

Percentage values were calculated using the total number of patients for whom data were available as the denominator. Data are given as mean ± SD, *n* (%) and median (interquartile range). GCS, Glasgow Coma Scale; ICH, intracerebral haemorrhage; IQR, interquartile range; mRS, modified Rankin scale; NIHSS, National Institutes of Health Stroke Scale; WMC, white matter changes.

### Associations of ‘early’ versus ‘late’ death

Of the 306 deaths, 156 occurred within 6 months of the index haemorrhage event (‘early’) and 150 deaths occurred after 6 months and within 3 years of the index ICH (‘late’). Baseline characteristics for both groups are shown in Table 1.

Early death (Table 2) was associated with age, hypertension, diabetes mellitus, atrial fibrillation, a history of previous cerebral ischaemic events, pre‐event modified Rankin scale (mRS) score, anticoagulant use prior to ICH, Glasgow Coma Scale (GCS) and National Institutes of Health Stroke Scale (NIHSS) scores at study entry. Imaging features at study entry that were significantly associated with early death were Van Swieten score, ICH volume and the presence of intraventricular extension. In a multivariable model including these variables, age at study entry (per year increase; HR, 1.05; 95% CI, 1.02–1.08, *P* = 0.003), history of hypertension (HR, 1.89; 95% CI, 1.04–3.46, *P* = 0.038), pre‐event mRS score (per point increase; HR, 1.41; 95% CI, 1.17–1.70, *P* < 0.0001), admission NIHSS score (per point increase; HR, 1.11; 95% CI, 1.06–1.15, *P* < 0.0001) and ICH volume >60 mL (HR, 4.08; 95% CI, 1.85–8.96, *P* < 0.0001) remained associated with early death.

**Table 2:** Univariable Cox regression analysis for early (before 6 months) and late (after 6 months) periods following intracerebral haemorrhage (ICH)

	‘Early’	‘Late’	Time‐varying coefficient *P*‐value
Age (per year increase)	1.08 (1.06–1.10)	1.09 (1.07–1.11)	0.360
Sex, male	0.73 (0.53–1.00)	0.85 (0.62–1.18)	0.500
Hypertension	1.43 (1.00–2.04)	1.08 (0.77–1.52)	0.270
Hypercholesterolaemia	1.18 (0.86–1.63)	1.34 (0.97–1.85)	0.585
Diabetes mellitus	1.44 (1.00–2.07)	1.31 (0.89–1.94)	0.729
Atrial fibrillation	2.31 (1.66–3.21)	2.67 (1.92–3.71)	0.548
Smoking, current	0.70 (0.39–1.27)	0.45 (0.22–0.91)	0.337
Pre‐existing cognitive impairment	1.32 (0.75–2.34)	2.13 (1.39–3.25)	0.336
Previous cerebral ischaemic event	1.50 (1.05–2.13)	1.90 (1.34–2.69)	0.345
Previous ICH	1.65 (0.87–3.14)	1.49 (0.73–3.04)	0.833
Pre‐event mRS score (per point increase)	1.56 (1.40–1.74)	1.50 (1.33–1.69)	0.610
*APOE* ε2, presence	1.40 (0.93–2.11)	0.70 (0.44–1.13)	0.032
*APOE* ε4, presence	0.65 (0.42–1.02)	0.90 (0.61–1.32)	0.280
Medications			
Antiplatelet use prior to ICH	0.98 (0.68–1.42)	0.96 (0.66–1.40)	0.936
Anticoagulant use prior to ICH	1.97 (1.44–2.71)	2.73 (1.97–3.78)	0.164
Antiplatelet use at discharge	0.95 (0.46–1.93)	1.23 (0.67–2.28)	0.582
Anticoagulant use at discharge	0.85 (0.49–1.48)	1.48 (0.93–2.35)	0.134
Clinical features at study entry			
GCS score (per point increase)	0.80 (0.76–0.84)	0.93 (0.86–1.00)	0.001
NIHSS score (per point increase)	1.11 (1.08–1.14)	1.00 (0.97–1.04)	<0.0001
Imaging features at study entry			
Lacunes, presence	1.05 (0.62–1.79)	1.06 (0.61–1.84)	0.976
Van Swieten score (WMC, per point increase)	1.23 (1.11–1.36)	1.37 (1.23–1.51)	0.139
ICH location			
Infratentorial	Reference group		
Deep	1.04 (0.57–1.93)	0.79 (0.47–1.32)	0.494
Lobar	1.42 (0.77–2.62)	0.67 (0.39–1.14)	0.067
ICH volume			
<30 mL	Reference group		
30–60 mL	2.20 (1.41–3.42)	1.29 (0.75–2.20)	0.131
>60 mL	4.85 (3.01–7.83)	1.40 (0.62–3.18)	0.010
Intraventricular extension	2.20 (1.61–3.02)	1.55 (1.11–2.18)	0.141

Univariable hazard ratios (HRs) for each characteristic obtained by fitting Cox regression models with time‐varying effects (before/after 6 months). Data are given as HR (95% confidence intervals). The time‐varying coefficient *P*‐value compares the difference between the early and late HRs. GCS, Glasgow Coma Scale; mRS, modified Rankin scale; NIHSS, National Institutes of Health Stroke Scale.

When considering death later than 6 months (Table 2), age, atrial fibrillation, smoking, pre‐event cognitive impairment, previous cerebral ischaemic event, pre‐event mRS score, anticoagulant use prior to index ICH, increasing van Swieten score and the presence of intraventricular extension showed significant associations. In a multivariable model including all variables with a significant association with late death, only age at study entry (per year increase; HR, 1.04; 95% CI, 1.02–1.08, *P* = 0.003), pre‐event mRS score (per point increase; HR, 1.42; 95% CI, 1.16–1.73, *P* = 0.001), anticoagulant use prior to ICH (HR, 2.13; 95% CI, 1.08–4.17, *P* = 0.028) and the presence of intraventricular extension (HR, 1.73; 95% CI, 1.05–2.85, *P* = 0.033) remained associated with late death.

We then investigated which baseline characteristics showed a significant change in HR between the early and late periods (Table 2). We found that HRs for the presence of *APOE* ε2, GCS score, NIHSS score and ICH volume >60 mL showed evidence of significant change between the early and late periods (Table 2).

### Further exploratory analysis of time‐varying effects

We then performed exploratory analyses where time was considered as a continuous measure (Table S1). In these analyses, variables that showed significant time‐varying effects were history of a previous cerebral ischaemic event (*P* = 0.0214), admission GCS score (*P* = 0.0108), NIHSS score (*P* < 0.00001) and ICH volume (*P* = 0.0439). The HRs of previous cerebral ischaemic events increased with time, whereas those for NIHSS score and ICH volume decreased with time. The protective (negative association) of GCS score also decreased with time.

## Discussion

We provide new evidence that not all baseline factors associated with early mortality after ICH are associated with mortality after 6 months. In analyses where the time‐varying effects of baseline variables were allowed to vary continuously with time, we found that the influence of measures of acute ICH severity decreased over time, whereas those associated with established cerebrovascular disease (previous cerebral ischaemic events) increased over time. These results support the argument that definitions of ‘early’ or ‘late’ death are necessarily arbitrary, as the impact of some characteristics present at study entry vary continuously with time.

In our study, the factors that we found to be independently associated with early death are in keeping with other studies and reflected in pre‐existing prognostic scores that include these and other variables [4, 5, 6, 7, 8, 9, 10, 11]. Differences between our results for associations with late death and those previously reported [14, 15] are likely to reflect our method of considering early and late death independently; when considering all death events together, we observed effects that were similar to those previously reported. Additionally, we observed that four variables (*APOE* ε2, GCS score, NIHSS score and ICH volume >60 mL) showed significant differences in the magnitude of their effect before and after 6 months (although the HRs for *APOE* ε2 were not statistically significant in themselves). This result confirms that, whereas GCS score, NIHSS score and ICH volume are important predictors of early mortality, their effect changes significantly between the early and late periods, and thus they are less useful for predicting mortality in the longer term, as we hypothesized. Our analyses of linear time‐varying effects on long‐term mortality following ICH are novel and demonstrate the potentially complex interactions that can occur over time. These analyses highlight the difficulties in defining what is ‘early’ death or a ‘short‐term’ outcome; further work that considers time‐varying effects on mortality across longer time scales is needed to guide this.

Our finding of an association between intraventricular extension and late death seems counterintuitive, but illustrates the importance of our work and the complicated manner in which baseline variables might interact over time. Given that our cohort included patients with milder strokes, we hypothesize that the effect of intraventricular extension on early death was lost in the adjusted analyses because of larger magnitude effects associated with other factors associated with ICH severity (NIHSS score and ICH volume). We speculate that, when considering late death, the effects of acute factors such as NIHSS score and ICH volume were of smaller magnitude, and thus the impact of intraventricular extension as a measure of stroke severity became more apparent. We did observe, in unadjusted analyses, that intraventricular extension was associated with both early and late death, but the magnitude of the association was smaller for late death (Table 2).

Our study has a number of strengths, including the number of patients, its multicentre design (which increases generalizability), robust ascertainment of follow‐up events and the detailed clinical and radiological data available for each participant. However, there are limitations of our work. Our cohort is comprised of survivors with mild strokes, as reflected by the median NIHSS and GCS scores, low ICH volumes and low early death rates. This cohort is therefore unlikely to be representative of all patients with ICH, particularly those with more severe haemorrhages. We were unable to adjust for acute complications of ICH or details relating to immediate care, either active or care‐limiting (i.e. do not resuscitate orders or palliative pathways), all of which would impact mortality. Additionally, we were unable to comment on cause of death in our patients. Finally, although we considered the time‐varying effects of variables recorded at study entry, the status of these may have changed after this time point (e.g. antiplatelet or anticoagulant use) and this could have influenced our results.

We provide new evidence that not all baseline factors associated with early mortality after ICH are associated with mortality after 6 months. Our findings could help design better prognostic scores for later death after ICH.

## Disclosure of conflicts of interest

H.C. reports grants and other support from Bayer Healthcare and UCB outside the submitted work. T.Y. reports personal fees and other support from GlaxoSmithKline, Biogen Idec, Novartis, ESOR, Merck, Hikma and Parexel outside the submitted work. G.Y.H.L. reports consultancy and speaker fees from Bayer, Bayer/Janssen, BMS/Pfizer, Biotronik, Medtronic, Boehringer Ingelheim, Microlife, Roche and Daiichi‐Sankyo outside the submitted work; no fees are directly received personally. K.W.M. reports personal fees from Bayer, personal fees and non‐financial support from Boehringer Ingelheim and personal fees from Daiichi‐Sankyo outside the submitted work. D.J.W. reports personal fees from Bayer, Alnylam and Portola outside the submitted work. The remaining authors declare no financial or other conflicts of interest.

## Supporting information


**Table S1. **Hazard ratios for variables with a significant time‐varying effect, at time 0 (study entry) and then 1, 2 and 3 years subsequently.

## Appendix 1

### The CROMIS‐2 collaborators

Louise Shaw MD, Kirsty Harkness MD, Jane Sword MD, Azlisham Mohd Nor MD, Pankaj Sharma PhD, Deborah Kelly MD, Frances Harrington MD, Marc Randall MD, Matthew Smith MD, Karim Mahawish MD, Abduelbaset Elmarim MD, Bernard Esisi MD, Claire Cullen MD, Arumug Nallasivam MD, Christopher Price MD, Adrian Barry MD, Christine Roffe MD, John Coyle MD, Ahamad Hassan MD, Caroline Lovelock DPhil, Jonathan Birns MD, David Cohen MD, L. Sekaran MD, Adrian Parry‐Jones PhD, Anthea Parry MD, David Hargroves MD, Harald Proschel MD, Prabel Datta MD, Khaled Darawil MD, Aravindakshan Manoj MD, Mathew Burn MD, Chris Patterson MD, Elio Giallombardo MD, Nigel Smyth MD, Syed Mansoor MD, Ijaz Anwar MD, Rachel Marsh MD, Sissi Ispoglou MD, Dinesh Chadha MD, Mathuri Prabhakaran MD, Sanjeevikumar Meenakishundaram MD, Janice O'Connell MD, Jon Scott MD, Vinodh Krishnamurthy MD, Prasanna Aghoram MD, Michael McCormick MD, Paul O’Mahony MD, Martin Cooper MD, Lillian Choy MD, Peter Wilkinson MD, Simon Leach MD, Sarah Caine MD, Ilse Burger MD, Gunaratam Gunathilagan MD, Paul Guyler MD, Hedley Emsley MD, Michelle Davis MD, Dulka Manawadu MD, Kath Pasco MD, Maam Mamun MD, Robert Luder MD, Mahmud Sajid MD, Ijaz Anwar MD, James Okwera MD, Julie Staals PhD, Elizabeth Warburton MD, Kari Saastamoinen MD, Timothy England MD, Janet Putterill MD, Enrico Flossman MD, Michael Power MD, Krishna Dani MD, David Mangion MD, Appu Suman MD, John Corrigan MD, Enas Lawrence MD and Djamil Vahidassr MD.
