# Supplementary material for: Baseline factors associated with early and late death in intracerebral haemorrhage survivors
Source: Eur J Neurol. 2020 Apr 28;27(7):1257–63. doi: 10.1111/ene.14238 (PMC7643267; doi:10.1111/ene.14238)
Supplement: Supplementary file 1 — Table S1. Hazard ratios for variables with a significant time‐varying effect, at time 0 (study entry) and then 1, 2 and 3 years subsequently. [file ENE-27-1257-s001.docx]

**SUPPLEMENTARY METHODS**

**Participants**

Participants were adults with spontaneous ICH confirmed on brain imaging (CT or MRI) within the preceding month. Patients with capacity gave informed written consent; in those without capacity, written consent was obtained from a proxy. as defined by relevant local legislation.

Patients were considered to have pre-existing cognitive impairment if they had a formal diagnosis of dementia or cognitive impairment at study entry, or if they scored more than 3.3 on the 16-item IQCODE (Informant Questionnaire for Cognitive Decline in the Elderly), in accordance with previous data(1). *APOE* genotype was established from peripheral blood samples; the method for this has been previously described(2).

**Imaging**

Imaging analysis was carried out by a clinical research associate and an MSc student, both of whom were trained in neuroimaging rating and blinded to the participant clinical details. Haematoma location was classified using the CHARTS (Cerebral Haemorrhage Anatomical RaTing inStrument) scale(3) as lobar (including convexity subarachnoid haemorrhage), or non-lobar (deep or brainstem haemorrhage); cerebellar haemorrhage was excluded from this definition as this does not have a clear small vessel disease association. CT images were also rated for the presence of lacunes, which were defined in accordance with consensus criteria(4). White matter changes (WMC) were rated using the Van Swieten score; the highest scores for anterior and posterior regions were combined in order to generate a “total” score (range 0 to 4)(5). Haematoma volume was rated using a semi-automated planimetric method, which has previously been described(6, 7). Intraventricular (IV) extension was defined as the presence of any blood within the cerebral ventricular system.

#### Statistics: Further exploratory analysis of time-varying effects

We performed further analyses, where the effect of each baseline variable was then allowed to vary linearly with time. Likelihood ratio tests (LRT) were used to evaluate the difference between the constant hazard ratio and the time-varying hazard ratio; values <0.05 were considered as significant (equivalent to violating the proportional-hazards assumption, which assumes that that the hazard ratio is constant over time). For those variables with a significant time-varying effect, we then calculated hazard ratios at 1-year intervals (i.e. at study entry, time 0, and then 1 year, 2 years and 3 years) to assess how the hazard ratio varies with time.

**SUPPEMENTARY RESULTS**

#### Further exploratory analysis of time-varying effects

We performed exploratory analyses where, instead of dichotomising time following ICH into “early” (before 6 months) and “late” (after 6 months) periods, time was considered as a continuous measure. In these analyses, variables which showed significant time-varying effects were history of a previous cerebral ischaemic event (p=0.0214), admission GCS (p=0.0108), NIHSS (p<0.00001) and ICH volume (p=0.0439). These models were then used to derive HR for each variable at study entry, and then 1 year, 2 years and 3 years following this; the time-varying effects of these variables are shown in Supplementary Table 1. The hazard ratios of previous cerebral ischaemic events increased with time, whilst those for NIHSS and ICH volume decreased with time. The protective (negative association) of GCS also decreased with time.

Supplementary Table 1: Hazard ratios for variables with a significant time-varying effect, at time 0 (study entry), and then 1 year, 2 years and 3 years subsequently

Univariable hazard ratios evaluated at various times for each characteristic obtained by fitting Cox regression models with linear time-varying effects.

|  | | **Study entry (time 0), HR (95% CI)** | **1 year after ICH,**  **HR (95% CI)** | **2 years after ICH,**  **HR (95% CI)** | **3 years after ICH,**  **HR (95% CI)** |
| --- | --- | --- | --- | --- | --- |
| Previous cerebral ischaemic event | | 1.26 (0.88 to 1.81) | 1.71 (1.33 to 2.19) | 2.32 (1.62 to 3.32) | 3.15 (1.77 to 5.59) |
| GCS, per point increase | | 0.81 (0.77 to 0.85) | 0.86 (0.82 to 0.90) | 0.91 (0.84 to 0.99) | 0.97 (0.86 to 1.10) |
| NIHSS, per point increase | | 1.11 (1.08 to 1.14) | 1.05 (1.03 to 1.08) | 1.00 (0.96 to 1.04) | 0.95 (0.89 to 1.01) |
| ICH volume | <30ml | *Reference group* | *Reference group* | *Reference group* | *Reference group* |
|  | 30 – 60ml | 2.14 (1.35 to 3.38) | 1.66 (1.16 to 2.36) | 1.28 (0.71 to 2.32) | 0.99 (0.39 to 2.56) |
|  | >60ml | 4.69 (2.80 to 7.84) | 2.59 (1.59 to 4.20) | 1.43 (0.57 to 3.59) | 0.79 (0.18 to 3.38) |

Abbreviations: CI, confidence interval; GCS, Glasgow Coma Scale; HR, hazard ratio; ICH, intracerebral haemorrhage; NIHSS, National Institutes of Health Stroke Scale; WMC, white matter changes.

**REFERENCES**

1. Harrison JK, Fearon P, Noel-Storr AH, McShane R, Stott DJ, Quinn TJ. Informant Questionnaire on Cognitive Decline in the Elderly (IQCODE) for the diagnosis of dementia within a secondary care setting. Cochrane Database Syst Rev. 2015;3(3):CD010772.

2. Crook R, Hardy J, Duff K. Single-day apolipoprotein E genotyping. Journal of neuroscience methods. 1994;53(2):125-7.

3. Charidimou A, Schmitt A, Wilson D, Yakushiji Y, Gregoire SM, Fox Z, et al. The Cerebral Haemorrhage Anatomical RaTing inStrument (CHARTS): Development and assessment of reliability. Journal of the neurological sciences. 2017;372:178-83.

4. Wardlaw JM, Smith EE, Biessels GJ, Cordonnier C, Fazekas F, Frayne R, et al. Neuroimaging standards for research into small vessel disease and its contribution to ageing and neurodegeneration. The Lancet Neurology. 2013;12(8):822-38.

5. van Swieten JC, Hijdra A, Koudstaal PJ, van Gijn J. Grading white matter lesions on CT and MRI: a simple scale. J Neurol Neurosurg Psychiatry. 1990;53(12):1080-3.

6. Volbers B, Staykov D, Wagner I, Dorfler A, Saake M, Schwab S, et al. Semi-automatic volumetric assessment of perihemorrhagic edema with computed tomography. Eur J Neurol. 2011;18(11):1323-8.

7. Wilson D, Charidimou A, Shakeshaft C, Ambler G, White M, Cohen H, et al. Volume and functional outcome of intracerebral hemorrhage according to oral anticoagulant type. Neurology. 2016;86(4):360-6.
